# Healthcare Costs and Health Status: Insights from the SHARE Survey

**DOI:** 10.3390/ijerph20021418

**Published:** 2023-01-12

**Authors:** Małgorzata Cygańska, Magdalena Kludacz-Alessandri, Chris Pyke

**Affiliations:** 1 Department of Finance, Economics and Finance Institute, The Faculty of Economics University o Warmia and Mazury in Olsztyn, 10-719 Olsztyn, Poland; 2 The College of Economics and Social Sciences, Warsaw University of Technology, 09-400 Plock, Poland; 3 Lancashire School of Business and Enterprise, University of Central Lancashire, Preston PR1 2HE, UK

**Keywords:** health status, cost outliers, older people, inpatient costs

## Abstract

The substantial rise in hospital costs over recent years is associated with the rapid increase in the older age population. This study addresses an empirical gap in the literature concerning the determinants of high hospital costs in a group of older patients in Europe. The objective of the study is to examine the association of patient health status with in-hospital costs among older people across European countries. We used the data from the Survey of Health, Ageing and Retirement in Europe (SHARE) database. The analysis included 9671 patients from 18 European countries. We considered socio-demographic, lifestyle and clinical variables as possible factors influencing in-hospital costs. Univariate and multivariable logistic regression analyses were used to determine the determinants of in-hospital costs. To benchmark the hospital costs across European countries, we used the cost-outlier methodology. Rates of hospital cost outliers among older people varies from 5.80 to 12.65% across Europe. Factors associated with extremely high in-patient costs differ among European countries. In most countries, they include the length of stay in the hospital, comorbidities, functional mobility and physical activity. The treatment of older people reporting heart attack, diabetes, chronic lung disease and cancer are more often connected with cost outliers. The risk of being a cost outlier increased by 20% with each day spent in the hospital. We advocate that including patient characteristics in the reimbursement system could provide a relatively simple strategy for reducing hospitals’ financial risk connected with exceptionally costly cases.

## 1. Introduction

The substantial increases in healthcare costs over recent years are associated with the rapid rise in the older population, greater longevity and the illness burden of older patients, who represent the fastest-growing age group in most developed countries [1]. Throughout developed European countries, life expectancy, together with the share of older people in society, is continuously increasing. The reason for this can be attributed to the success of both preventive and curative medicine, combined with widespread technological advances and declining fertility rates [2]. It is widely known that older people generally use more health services than other age groups, are at higher risk of more expensive long-term institutional care and consume the majority of health care expenditure. An ageing population, together with the increasing cost of medical technologies, has been indicated as a significant cause of the increase in healthcare expenditures and places a particular burden on hospital medical services. Hospital services are the most expensive component of health systems, and hospital in-patient care represents the most significant single component of healthcare expenditures [3]. It was observed that the average per-capita total healthcare expenditure is higher for older patients, mainly due to in-patient hospital-related spending. Older patients represent a large number of hospital admissions, consuming a substantial share of hospital resources. They will often increase the demand for acute beds and have a prolonged hospital stay [4]. Limited financial resources allocated for hospitalised older patients represent a significant economic problem for public health systems and their families. European countries must prepare their healthcare systems for an ageing population. Thus, the question is what are the most important determinants of high hospital costs of elderly patients and whether these determinants are similar across Europe.

A high proportion of hospital costs incurred at the end of life are usually associated with managing terminal illness [5] and the type of care received at this time [6]. However, it should be noted that the link between population ageing and the level of hospital costs might be more complicated [7]. Some studies suggest that the most important determinants of hospital expenditure on elderly patients seem to be those connected with age-related impairments (e.g., cognitive impairment, falls, fractures) and with chronic diseases (e.g., cancer, stroke, heart disease, lung disease) [8,9]. Other authors have shown a greater probability of high in-patient costs with complications such as a readmission, an emergency admission, a higher severity score within the Diagnosis Related Groups (DRG) and death during hospitalisation [10]. Therefore, one of the essential determinants of hospital costs for the elderly is their state of health. On the other hand, Meerding et al. [11] point out that the relationship between disease and costs is not straightforward, and relevant data is often lacking to conduct an analysis. These authors also tried to determine which illnesses, sex and age groups have the greatest demand for care and therefore the highest total healthcare costs.

As the average age of the population increases, so does the prevalence of chronic diseases, which are considered the most significant threat to health status and the largest cause of healthcare expenditures. Chronic diseases cause increasing numbers of deaths worldwide. They are responsible for almost 70% of health care expenditure [12]. A substantial healthcare problem, especially among the elderly, is multiple chronic conditions (also called multi-morbidity). The prevalence of multiple chronic conditions among people aged 65 and older has been widely reported to exceed 65% [2]. The reality is that 50% of all patients with chronic conditions have multiple chronic conditions. This situation leads to a more rapid decline in health status as seniors may be more susceptible to treatment complications due to physical frailty, complicated drug regime and poor coordination of care [13]. On the other hand, an earlier diagnosed chronic illness indicates previous use of services and leads to further contacts for monitoring and cheaper treatment. 

According to some researchers, the most critical determinants of hospital expenditure are initial disease severity and the clinical course followed by the patient during hospitalisation [14]. These factors are significant for the costs of treatment of elderly patients because of an intensification of comorbidities and complications in older age. The comorbidities prolong the length of hospital stay (LOS), which can also increase hospitalisation’s direct costs [3,6]. Information on the determinants of hospital expenditures amongst older people is one of the most important issues in health policy. 

The difference in the determinants of in-patients costs can be associated with the patient’s health status and the healthcare service quality, complications rate, and organisation of the healthcare delivery system in European countries. 

Special funding for cost outliers, beside separate payments and supplementary payments, is one of the three main short-term payment instruments used by different countries aiming to incentivize hospitals to adopt and use technological innovation. In countries where special funding for cost outliers is available (e.g., Sweden, Poland, Estonia, Finland, Switzerland), the way technologies influence the homogeneity of resource use of cases within DRGs determines whether special funding is made available on top of standard payment rates. Cost-outlier funding builds on a detailed retrospective statistical analysis of cost data. However, most European countries finance outliers based on a per diem charge over a certain length of stay threshold, as cost data are not available for all cases. 

According to some authors, hospital cost outliers account for almost 18% of hospital costs [15]. Despite their importance, research on the factors that impact hospital high-cost outliers of an ageing population is scarce. Cost-outlier methodology allows us to compare the in-hospital costs in different countries with different healthcare expenditures. Revealing exceptional behaviors of certain patients’ costs is important since it signifies that some extraordinary circumstances have occurred. Additionally, hospitals pursue the reduction of the cost of hospital stays using a rational management strategy. Accordingly, high-cost outliers are of great interest to managers but also shall deserve the attention of doctors. The determination of outliers is important in developing the country and regional health policy. As the hospitals try to moderate the consequences of case-mix financing systems in many countries, in the longer term, they seek to have clinically justifiable outliers funded on the basis of their real cost.

The aim of our study is to indicate the impact of health status on in-hospital costs among older people across European countries. In this paper, we describe and compare the characteristics of elderly patients with regular (average) hospital costs (cost inliers—CI) and elderly patients with extremely high hospitalisation costs—cost outliers (CO), defined as a data object that does not comply with the general behaviour and is very different and inconsistent with the remaining data [16]. We identify socio-economic, clinical and medical factors associated with hospital CO. 

## 2. Materials and Methods

### 2.1. Sample and Data

The data used in this study is from the Survey of Health, Ageing and Retirement in Europe (SHARE) database. It is a multidisciplinary, cross-national micro database containing information on the health, social networks and socioeconomic status of over 140,000 people aged 50+ from 28 European countries and Israel. The SHARE database offers considerable opportunities for researchers because it enables them to study patient-level data. Data collection is based on computer-assisted personal interviewing (CAPI). Hundreds of variables are available in this dataset, including information on demographics, social networks, physical and mental health, behavioural risks, cognitive functions, healthcare, employment and pensions, income, social support, consumption, assets, financial transfers and many other factors.

The SHARE target population consists of all persons aged 50 years and over at the time of sampling who have their regular domicile in the respective SHARE country. Persons are excluded from baseline or refreshment samples if they are incarcerated, hospitalised or out of the country during the entire survey period; unable to speak the country’s language(s); or have moved to an unknown address.

In this paper, we used cross-sectional data from the 6th wave of the survey, which was completed in 2015 [17]. From 68,231 SHARE respondents, 10,394 persons claimed to spend at least one night in the hospital in 2014. We excluded from analysis 723 cases because of missing data. Finally, the sample includes 9671 elderly people, aged 50+, (from the following countries: Austria, Belgium, Czech Republic, Denmark, Estonia, France, Germany, Greece, Italy, Luxembourg, Poland, Portugal, Slovenia, Spain, Sweden and Switzerland [18]. 

Our analysis looks at the elderly who claimed to spend at least one night in the hospital in the previous year. It is focused on self-reported health outcomes and objective measures characterising the social, demographic and clinical situation of individual older people and the costs of their hospitalisation. These costs were calculated based on information from the OECD database regarding expenditures on in-patient curative and rehabilitative care provided by hospitals per capita in current prices divided by the average length of stay in the country’s hospitals. All costs were converted into Euro values.

### 2.2. Variables

The statistical analysis was performed in steps. First, explanatory variables were chosen according to theoretical considerations, data availability and statistical criteria. We considered socio-demographic, lifestyle and clinical variables as the possible factors influencing the hospital’s high-cost outliers. Socio-demographic variables included gender, age and job situation. This data came from the cover screen—the first module of the SHARE Interviewer Survey—and the job situation data came from the Employment & Pensions (E.P.) module, including information about the respondent’s current work activities.

Clinical variables covered the length of stay per hospitalisation (LOS) and the number of nights spent in the hospital during the last twelve months, as well as health risk factors, health status and functional ability. Health risk factors included B.M.I., high blood cholesterol, hypertension, physical activity and previous heart attack. Functional ability factors considered physical and mental conditions, including difficulties with walking 100 m and memory abilities. Health status factors looked at several chronic diseases and the medical diagnoses and comorbidities, such as stroke, cerebral vascular disease, cancer, chronic lung disease, diabetes and high blood sugar.

This information came from the health module of the SHARE questionnaire, which contains a broad range of physical and mental health measures and indices from six different CAPI modules: physical health (P.H.), behavioural risks (B.R.), cognitive function (C.F.), mental health (M.H.), grip strength (G.S.) and walking speed (W.S.). The information about memory came from the health module “ten-word-list learning test”, which is conducted with a first trial and a delayed recall.

We calculated the individuals’ hospitalisation costs by multiplying the total number of nights spent in the hospital in 2015 for each patient with the average in-patient cost per day for the country. The in-patient cost per day was calculated by dividing hospital expenditure (in-patient curative and rehabilitative care costs in 2015) in evaluated countries by the number of in-patient curative bed days in each country based on information from the OECD database.

### 2.3. Statistical Analysis

Descriptive statistics for patient characteristics were based on percentages and frequencies for qualitative variables and for continuous variables. Means and SDs were calculated for all data. Medians and interquartile ranges (Q1–Q3) were also used if the data were skewed. For instance, calculations of the LOS., total nights in the hospital and costs are indicated with both a mean and a median. Thus the best-estimated costs and duration of stay per patient and typical patient are reflected. Qualitative and continuous variables were compared using the Chi-squared test. Student’s t-test or Mann–Whitney U-test was used to compare the variables if data was skewed. The means and standard deviations (SD) were calculated for costs. 

We used the interquartile method to select the outliers using the median and the interquartile distance. The high outliers were identified as the 75th percentile +1.5* interquartile range. We used the corresponding rule: 25th percentile—1.5* interquartile scope to determine the low-cost outliers. Because the rule 25th percentile—1.5* interquartile range detected a negative trim-point in further analysis, we considered only high-cost outliers. The study of contingency tables was executed with Pearson’s χ2-test. Continuous variables without normal distribution were analysed with the Mann–Whitney test. Univariate and multivariate logistic regression analysis was used to identify the determinants of CO. Being a high-cost outlier was the dependent variable in the logistic regression analysis. A *p*-value of 0.005 was considered significant. Statistical analysis was carried out using StatSoft Statistica software version 13.3.

## 3. Results

Socio-demographic and health characteristics of the population studied are presented in Table 1. The patients included in the analysis were more likely to be retired (67.9%). The majority of them were female (53.9%). The average age was 58 years. Overall average LOS was 7.23 days. The average number of chronic diseases per patient was 2.65. The most frequently found chronic disease (hypertension) affected more than 50.5% of patients, followed by diabetes (20.1%) and chronic lung disease (12.3%). A total of 25% of patients had difficulty walking 100 m, and 23% suffered from a heart attack.

Table 2 presents hospital cost outliers (CO) and inliers (CI) by country. In the study, 873 patients were identified as cost outliers. The highest share of CO was in Switzerland (12.7%) and the lowest in Italy (5.8%). The lowest percentage of outliers was observed the in the countries of south and middle Europe (Germany, Czech Republic, Austria, Italy, Greece and Estonia). The highest was observed in the countries of west Europe and Poland.

**Figure 1:**
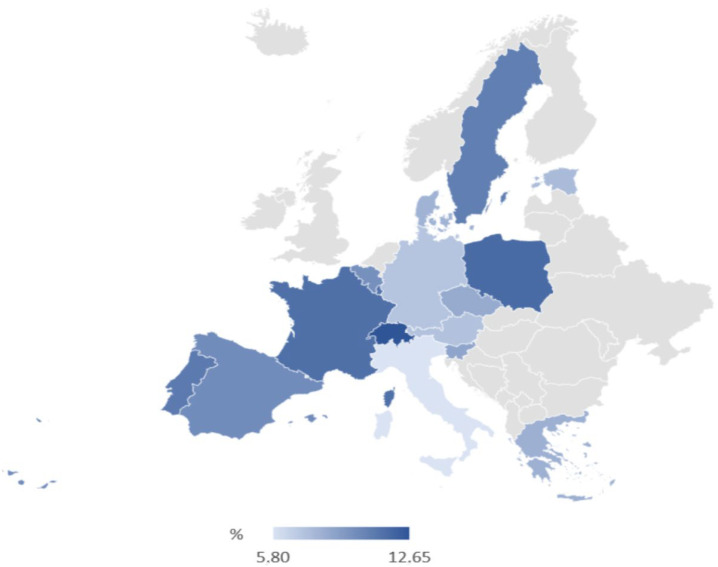
Geographical distribution of % of hospital cost outliers relative to all hospitalised patients.

The geographical distribution of % of hospital cost outliers relative to all hospitalised patients is present in Figure 1.

The cost outliers (CO) and cost inliers (CI) according to patient socio-demographic and health characteristics are compared in Table 3. 

The highest difference between inliers’ and outliers’ costs was observed in Portugal, where outliers’ costs were more than 14 times higher than inliers’ costs. This difference was associated with four patient characteristics (LOS, BMI, NH, Gender). The significant difference between inliers’ and outliers’ patient characteristics was also observed in Belgium, Estonia, Spain, France and Denmark (more than 8). The lowest difference was observed in Greece, with outlier costs higher six times compared to inlier costs and with five patient characteristics statistically significantly differentiating in-patient and outpatient costs (LOS; NH; DW; SV; Cancer). The minor difference between outliers’ and inliers’ costs was also observed in Poland, Switzerland and Austria.

We analysed all countries using univariate linear regression models in the next step. We used a univariate logistic regression with all the variables, and the odds ratios (ORs) for each variable are presented in Table 4. Difficulties with walking 100 m significantly increased the cost of health care, having the highest OR coefficient. The second-highest coefficient was reached by the variable “Hardly ever, or never” regarding the number of vigorous activities in daily life, confirming the importance of sport activity in decreasing the cost of care. The number of chronic diseases influenced cost outliers (OR = 1.173, CI 95%: [1.134–1.214]). The kind of chronic diseases was also important. For “cancer”, OR was 2.029 (CI 95%: [1.683–2.446]); for “chronic lung disease”, OR was 1.540 (CI 95%: [1.276–1.859]); for “diabetes”, OR was 1.541 (CI 95%: [1.315–1.806]); for “stroke“, OR was 2.258 (CI 95%: [1.856–2.746]); that is, high-cost outliers were more likely to happen in these chronic diseases. On average, the LOS OR was 1.224 (CI95%: [1.210–1.239]) and, for the number of nights stayed in the hospital, 1.385 (CI 95%: [11.349–1.421]). The least-active people had significantly more high-cost outliers than more-active people (OR = 2.813, CI 95%: [2.269–3.486]). Furthermore, people with difficulties walking 100 m were clearly more likely to have high-cost outliers (OR = 3.305, CI 95%: [2.868–3.809]) compared to those without such problems. Regarding the job status, permanently sick or disabled individuals have more high-cost outliers than other groups (OR = 1.811, CI95%: [1.416–2.317]). Considering gender, we did not find significant differences between women and men. Women had an adjusted OR of 1.055 (not statistically significant, CI95%: [0.917–1.213]). We also found that factors such as age, BMI, hypertension and high blood cholesterol were unimportant. 

It is worth emphasizing that some of the health symptoms like BMI, hypertension and high blood cholesterol were unimportant in terms of cost outliers; however, we found out that patients with secondary diseases, like diabetes and stroke, needs extra financial resources for treatment (variables that are statistically significant). 

In order to deepen the analysis, since the univariate analysis concentrate on one variable was involved separately, we provided the multivariate analysis. It allows us to identify the relations among the measurements and identify their cumulative effects on being patient outliers. We included in the model all independent variables that were proven significant in the univariate analysis (Table 5). 

In multivariate analysis, we found that permanently sick or disabled individuals (OR = 1.654, [95% CI, 1.173–2.330]) and those who suffered from cancer (OR = 2.869 [95% CI, 2.232–3.688]), diabetes (OR = 1.353, [95% CI, 1.062–1.724]), heart attack (OR = 1.261, [95% CI, 1.003–1.586]) or chronic lung disease (OR = 1.540, [95% CI, 1.276–1.859]) were associated with being hospital cost outliers. Furthermore, individuals who reported difficulties with walking 100 m (OR = 1.771, [95% CI, 1.433–2.190]) are more often cost outliers. Moreover, the risk of being a cost outlier increases by 21.9% with each day spent in hospital (OR = 1219, [95% CI, 1.205–1.234]). 

## 4. Discussion

In this study, we examined if the patient health status is associated with hospital treatment costs among older people across European countries. We also analysed the impact of various socio-demographic and clinical factors on the high-cost outliers of elderly patients (CO) in Europe. Eight predictors of cost outliers are highlighted in this study. They are LOS, current job situation, the severity of illness, functional mobility and multiple chronic diseases. 

In general, our findings suggest that the most important determinants of CO were: disability, number of chronic diseases and some kinds of chronic diseases, especially cancer. One of the most important determinants of hospital cost outliers of older people is disability (permanently sick or disabled, with difficulties with walking 100 m.). The conclusion that functional disabilities are a strong determinant of healthcare costs among older people was confirmed by Wagner et al. [19]. Functional independence measure (FIM) scores were significantly associated with health costs. They explained that FIM affects discharge costs mainly through its association with LOS. They also found a strong relationship between LOS and in-patient rehabilitation costs. According to them, LOS explained approximately 80% of the variance in the cost of specialised in-patient rehabilitation stays because such costs are fixed or recurring. Another study conducted on patients with stroke showed that the patients more disabled at discharge and having longer LOS are more likely to incur higher costs of care [20]. It is worth drawing attention to the cost outliers (CO) of patients with a prolonged length of stay (PLOS), who are treated as a specific category of patients [15,21,22]. However, in our study, disability and functional activity turned out to be independent of LOS. These different results can be caused by the diverse study populations, especially in terms of age. This is consistent with other studies showing that LOS does not directly impact hospital costs. Other factors, like comorbidities, can prolong the length of hospital stays, increasing the direct costs of hospitalisation [3,6]. Some researchers have found that the correlation between LOS and hospital costs is strong but not perfect, so LOS can only partly explain hospital costs [23]. In Pirson’s study [24], LOS outliers and cost outliers were not connected with the same patients. A preliminary comparison of these patients showed that 32% of cost outliers have a normal LOS and that 48% of LOS outliers have a standard cost.

Our study revealed the relationship between the cost outliers and the number and kind of chronic diseases and impairments. Our findings are confirmed by Lenhert et al. [2] based on 35 studies concerning the relationship between multiple chronic diseases and health expenditure for ageing populations. They found a positive association between multiple chronic diseases and health expenditure. They noticed that health expenditure significantly increased with each additional chronic disease, and several of them observed a curvilinear (near exponential) relationship, in which expenditure roughly doubled with each additional chronic disease [13,25]. Generally, the more diseases a patient has, the higher the costs of additional diseases.

Folasade et al. [26] identified differences in healthcare utilisation and mortality by race/ethnicity. These findings may explain a higher number of patient characteristics that statistically significantly differentiate in-patient and outpatient costs factors. Segheri et al. [27] underlie the importance of considering the impact of the governance system as one of the possible determinants of the geographic variation of healthcare costs when comparing different regional healthcare systems, which is consistent with our study. 

Similar to other studies, we did not find age and gender to be associated with being high-cost outliers [20]. On the other hand, some other researchers reported that females were more likely to experience higher costs than males [28]. Our findings suggest that, although women had higher use of health services, they accounted for less hospital expenditure. It could be related to the fact that women are more aware of health matters than men and take better care of themselves [29]. Furthermore, they visit physicians more often than men, resulting in the earlier detection of health problems [30]. 

Some researchers found that lower physical activity levels in older adults are associated with higher public expenditure on healthcare [7]. In our research, the level of activity was not a statistically significant factor affecting CO. We presume that the individuals reported as high-cost outliers are significantly less active because they are permanently sick or disabled; have problems with walking; suffered from a heart attack; or have more chronic diseases, like cancer, diabetes or chronic lung diseases. Because of these problems, they are more often admitted to hospitals and spend more time there. It is unclear, however, whether the low activity of the elderly is the cause or the result of disability, which we believe is the main factor affecting the high costs of hospital treatment for the elderly. Therefore, discovering these relationships requires further research.

According to our analysis, older people with health problems, such as heart attack, diabetes, chronic lung disease or cancer generate the highest costs in hospitals. According to an American study, two-thirds of older Medicare beneficiaries had two or more types of chronic conditions and accounted for 99% of health expenditures, but the reason for this situation is a higher risk of hospital complications that increase the costs [31]. The study regarding older patients with pneumonia showed that neither patient age nor comorbidities were independently associated with high costs if they had no related complications [32]. Likewise, Kaplan et al. [33] also found patients with complications require more extended hospital stays, resulting in increased costs. The other authors found that complications increase the costs up to six times due to the need for more tests and treatments, especially due to LOS prolongation [32]. Older people with multiple chronic conditions are high users of hospital care, in particular emergency-department services, mainly because of higher LOS caused by frequent hospital admissions for adverse events [34].

While it is important not to confuse cost outliers and LOS outliers, LOS has an important impact on hospital costs. Each country should examine the explanatory factors that may be modified by clinicians and managers working together. In particular, efforts aimed at optimising the safe and dignified but timely discharge of patients should be more systematised than they are currently. A clear understanding of the drivers of the high in-patient costs of older patients is essential for developing and implementing policies to improve public expenditure efficiency. Outlier payment systems integrated with hospital financial systems could, for example, encourage hospitals to use technological innovations. We advocate that including patient characteristics in the reimbursement system could provide a relatively simple strategy for reducing hospitals’ financial risk connected with exceptionally costly cases.

The added value of our research is that we have analysed the costs connected with many chronic diseases and disabilities across European countries and identified the ones that have the highest risk of causing the high occurrence of in-patient cost outliers. In many other studies, social and clinical factors were statistically significant predictors of health costs, but they were not analysed using extensive international data on the ageing population. There is a lack of population-based empirical studies concerning the complex relationship between high-hospital-costs outliers, socio-demographic characteristics and the senior’s health status. Our study is the first using the SHARE database that provides important new figures from a comprehensive healthcare perspective on the complex relationship between hospital cost outliers and their’ potential determinants in a severely understudied and rapidly growing group of seniors in Europe. The limitation of other studies is underestimating the role of specific chronic diseases that make up a large part of healthcare costs. 

The strength of our analyses is derived from using a large and representative European dataset. In addition, unlike most studies, we did not concentrate on a specific hospital, country or individual patient under the same diagnosis. Still, we conducted a cross-national comparison of the determinants of hospital costs in European countries.

The main limitation of our study refers to limited number of measures and self-reported health status while the interview process. Therefore the results must be interpreted carefully. Future research, based on more detailed data, from medical documentation, could analyse the studied relations in a more detailed way. 

## 5. Conclusions

A better understanding of cost outliers is essential to improving the management and financing of hospitals and the whole healthcare system. The high-cost outliers are of particular interest to hospital managers who aim to reduce the cost of hospital stays using a rational management strategy and to find clinical justification for funding these outliers based on their actual costs in order to moderate the consequences of a case-mix financing system. Knowledge of cost outliers is also important for protecting more expensive patients and for the appropriate financing treatment of uncommon cases [35]. In an era of increasing budget restrictions, the economic analysis of cost outliers and identifying the factors affecting high health cost outliers could help define strategies for cost reduction or the more efficient use of existing resources. Special funding for cost outliers in some European countries is available to provide strong incentives for hospitals to use technological innovations [36]. The models to identify potential cost outliers are needed to design the outlier policy in the country. 

## Figures and Tables

**Table 1:** Demographic and health characteristics of the population studied (n = 9671).

Patients Characteristics	Mean (SD) Median [Q1–Q3]
Age [years]	57.81 (22.29)
Average LOS [days]	7.23 (12.47) 4.00 [2.00–8.00]
BMI	26.62 (6.84)
Nights stayed in hospital [per year]	11.74 (21.38) 6.00 [3.00–12.00]
Number of chronic diseases	2.65 (1.83)
Gender	n (%)
Female	5212 (53.88)
Male	4461 (46.12)
Job situation	
Retired	6572 (67.94)
Employed or self-employed	1325 (13.70)
Unemployed	188 (1.94)
Homemaker	537 (5.55)
Permanently sick or disabled	637 (6.58)
Other	191 (4.29)
Sports or activities that are vigorous	
More than once a week	2172 (22.45)
Once a week	946 (9.78)
One to three times a month	687 (7.10)
Hardly ever. or never	5868 (60.66)
Number of words recalled after learning	
0–2	796 (8.98)
3–6	6176 (69.67)
7–9	1864 (21.35)
Difficulties with walking 100 m	
Present	2404 (24.85)
Absent	7268 (75.14)
Heart attack	
Present	2254 (23.30)
Absent	7417 (76.68)
Hypertension	
Present	4872 (50.39)
Absent	4799 (49.61)
High blood cholesterol	
Present	2764 (28.60)
Absent	6907 (71.40)
Stroke	
Present	839 (8.67)
Absent	8832 (91.31)
Diabetes	
Present	1941 (20.09)
Absent	7730 (79.91)
Chronic lung disease	
Present	1186 (12.28)
Absent	8485 (87.72)
Cancer	
Present	1008 (10.44)
Absent	8863 (89.56)

SD—standard deviation; Q1—first quartile; Q3—third quartile.

**Table 2:** Hospital cost outliers (CO) and inliers (CI) by country.

Country	CI n = 8800	CO n = 873
	n (%)	
Austria	749 (92.58)	60 (7.41)
Belgium	892 (90.19)	97 (9.81)
Czech Republic	867 (91.45)	81 (8.54)
Denmark	441 (91.87)	39 (8.12)
Estonia	762 (92.25)	64 (7.74)
France	484 (88.64)	62 (11.35)
Germany	827 (92.71)	65 (7.29)
Greece	356 (91.75)	32 (8.25)
Italy	552 (91.20)	34 (5.80)
Luxembourg	230 (88.80)	29 (11.19)
Poland	297 (88.39)	39 (11.61)
Portugal	197 (89.14)	24 (10.86)
Slovenia	610 (91.18)	59 (8.81)
Spain	639 (90.00)	71 (10.00)
Sweden	496 (89.37)	59 (10.63)
Switzerland	401 (87.36)	58 (12.65)

**Table 3:** Characteristics of cost outliers (CO) and inliers (CI) by country.

Country	CI n = 8800	CO n = 873	Median Outpatient Costs/Median Inpatient Cost	Patient Characteristics Statistically Significantly Differentiate In-patient and Outpatient Costs Factors
	In-Patient Costs Median [Q1–Q3] [€/person]			
Austria	926.94 [463.47–1853.88]	6643.07 [6025.11–9269.40]	7.16	LOS; NH; ChD; DW; SV; Words; HA; Stroke; CLD; Cancer
Belgium	571.56 [142.89–1143.12]	5144.04 [4286.70–8573.40]	9.00	LOS; NH; ChD; DW; SV; JS; Words; HA; HBP; Stroke
Czech Republic	211.68 [120.96–393.12]	1814.40 [1209.60–2540.16]	8.57	LOS; NH; ChD; DW; SV; Words; Cancer
Denmark	756.15 [252.05–1512.30]	7561.50 [6049.20–11,342.25]	10.00	Age; LOS; NH; ChD; DW; SV
Estonia	183.54 [91.77–305.90]	1758.92 [1223.60–2753.10]	9.58	LOS; NH; DW; SV; Stroke
France	417.20 [208.60–834.40]	4484.90 [3129.00–6779.50]	10.75	LOS; NH; ChD; DW; SV; JS; Words; HA; Stroke
Germany	749.22 [374.61–1373.57]	6118.63 [4994.80–8241.42]	8.17	Age; LOS; NH; ChD; DW; SV; JS; HA; Stroke; Cancer
Greece	371.05 [222.63–742.10]	2226.30 [2226.30–3710.50]	6.00	LOS; NH; DW; SV; Cancer
Italy	848.52 [282.84–1555.62]	6929.58 [5373.96–8626.62]	8.16	Age; LOS; NH; ChD; DW; Words; Stroke; Cancer
Luxembourg	848.52 [282.84–1555.62]	6929.58 [5273.96–8626.62]	8.16	LOS; NH; DW; SV; Words
Poland	241.36 [137.92–448.24]	1586.08 [1413.68–2413.60]	6.57	LOS; NH; SV; Cancer
Portugal	118.04 [59.02–236.08]	1711.58 [885.30-2434.57]	14.5	LOS; BMI; NH; Gender
Slovenia	384.30 [192.15–640.50]	3202.50 [2690.10–3843.00]	8.33	LOS; NH; ChD; DW; SV; JS
Spain	351.80 [140.72–703.60]	3166.20 [2110.80–4925.20]	9.00	LOS; NH; DW; Stroke; Cancer
Sweden	546.27 [273.14–1092.54]	4370.16 [3459.71–6373.15]	8.00	LOS; BMI; NH; DW; SV; CLD; Cancer
Switzerland	1378.90 [551.56–2206.24]	9652.30 [7721.84–15,443.68]	7.00	Age; LOS; NH; ChD; DW; SV; JS; CLD

Q1—first quartile; Q3—third quartile. LOS—length of stay; NH—nights stayed in hospital; ChD—number of chronic diseases; DW—difficulties with walking 100 m; SV—vigorous sport; JS—current job situation; Words—number of words learning tests. HA—heart attack; HBP—high blood cholesterol; CLD—chronic lung disease.

**Table 4:** Univariate analysis of factors associated with cost outliers.

Patient Characteristics	OR	(95% CI)	*p*-Value^*^
Age [years]	1.003	(0.999–1.006)	NS
average LOS [days]	1.224	(1.210–1.239)	<0.001
BMI	0.979	(0.970–0.988)	NS
Nights stayed in hospital [per year]	1.385	(1.349–1.421)	<0.001
Number of chronic diseases	1.173	(1.134–1.214)	<0.001
Gender			
Female	1.055	(0.917–1.213)	NS
Male	1		
Current job situation			
Retired	1		
Employed or self-employed	0.269	(0.191–0.378)	<0.001
Unemployed	0.428	(0.210–0.873)	<0.001
Homemaker	0.892	(0.667–1.193)	NS
Permanently sick or disabled	1.811	(1.416–2.317)	<0.001
Other	2.034	(1.557–2.657)	<0.001
Vigorous sport			
More than once a week	1		
Once a week	0.718	(0.479–1.076)	NS
One to three times a month	1.001	(0.666–1.505)	NS
Hardly ever. or never	2.813	2.269–3.486)	<0.001
Number of words learning			
0–2	1		
3–6	0.496	(0400–0.615)	<0.001
7–9	0.334	(0.253–0.440)	<0.001
Difficulties with walking 100 m			
Absent	1		
Present	3.305	(2.868–3.809)	<0.001
Heart attack			
Absent	1		
Present	1.501	(1.289–1.749)	<0.001
Hypertension			
Absent	1		
Present	0.991	(0.862–1.139)	NS
High blood cholesterol			
Absent	1		
Present	0.931	(0.796–1.088)	NS
Stroke			
Absent	1		
Present	2.258	(1.856–2.746)	<0.001
Diabetes			
Absent	1		
Present	1.541	(1.315–1.806)	<0.001
Chronic lung disease			
Absent	1		
Present	1.540	(1.276–1.859)	<0.001
Cancer			
Absent	1		
Present	2.029	(1.683–2.446)	<0.001

**Table 5:** Multivariate analysis of factors associated with cost outliers.

Explanatory Variables	β-Coefficient	Adjusted		
		OR	(95% CI)	*p*-Value
LOS [days]	0.198	1.219	(1.205–1.234)	<0.001
Employed or self-employed	−0.688	0.502	(0.313–0.806)	0.004
Permanently sick or disabled	0.503	1.654	(1.173–2.330)	0.004
Difficulties with walking 100 m	0.572	1.771	(1.433–2.190)	<0.001
Heart attack	0.232	1.261	(1.003–1.586)	0.004
Diabetes	0.302	1.353	(1.062–1.724)	0.001
Chronic lung disease	0.176	1.540	(1.276–1.859)	<0.001
Cancer	1.054	2.869	(2.232–3.688)	<0.001

CI—confidence interval, OR—odds ratio, LOS—length of stay.

## Data Availability

This paper uses data from SHARE Wave 6 (DOIs:10.6103/SHARE.w6.800); see Börsch-Supan et al. (2013) for methodological details [37]. The SHARE data collection has been funded by the European Commission, DG RTD through FP5 (QLK6-CT-2001-00360), FP6 (SHARE-I3: RII-CT-2006-062193, COMPARE: CIT5-CT-2 005-028857, SHARELIFE: CIT4-CT-2006-028812), FP7 (SHARE-PREP: GA N°211909, SHARE-LEAP: GA N°227822, SHARE M4: GA N°261982, DASISH: GA N°283646) and Horizon 2020 (SHARE-DEV3: GA N°676536, SHARE-COHESION: GA N°870628, SERISS: GA N°654221, SSHOC: GA N°823782, SHARE-COVID19: GA N°101015924) and by D.G. Employment, Social Affairs & Inclusion through vs. 2015/0195, vs. 2016/0135, vs. 2018/0285, vs. 2019/0332 and vs. 2020/0313. Additional funding from the German Ministry of Education and Research, the Max Planck Society for the Advancement of Science, the U.S. National Institute on Aging (U01_AG09740-13S2, P01_AG005842, P01_AG08291, P30_AG12815, R21_AG025169, Y1-AG-4553-01, IAG_BSR06-11, OGHA_04-064, HHSN271201300071C, RAG052527A) and from various national funding sources is gratefully acknowledged (see www.share-project.org (accessed on 21 December 2022)).
